# Effect of Coriander Seed Addition at Different Stages of Brewing on Selected Parameters of Low-Alcohol Wheat Beers

**DOI:** 10.3390/molecules29040844

**Published:** 2024-02-14

**Authors:** Aneta Pater, Paweł Satora, Magdalena Januszek

**Affiliations:** Department of Fermentation Technology and Microbiology, Faculty of Food Technology, University of Agriculture, Balicka Street 122, 30–149 Kraków, Poland; magdalena.januszek@urk.edu.pl

**Keywords:** low-alcohol beers, coriander seeds, volatiles, odor-active compounds

## Abstract

In recent years, there has been a significant decline in interest in high-alcohol beers, while interest in low- and non-alcohol beers is growing. The aim of this study was to investigate the influence of the addition of coriander seeds at various stages of the production of low-alcohol wheat beer (mashing, boiling, and fermentation). The presented article uses biological methods to produce low-alcohol beer. For this purpose, first, the mashing process was modified (breaking 44 °C for 20 min, followed by 75 °C for 60 min). The chemical composition and aroma components of the obtained beers were determined using various chromatographic methods (HPLC, GC-MS, and GC-O). Differences were found between the aroma components depending on the stage of production at which the coriander seeds were added. Beers with the addition of coriander seeds at the fermentation stage had the highest terpene content (linalool, camphor, trans-linalool oxide, and γ-terpinene) and boiling (myrcene, limonene, citronellol, and geraniol). The least desirable process is the addition of coriander seeds at the mashing stage due to the lowest content of volatile compounds. Additionally, beers with the addition of coriander seeds for fermentation were characterized by a higher content of antioxidant compounds. This proves that the addition of coriander seeds during beer production could improve the fermentation process and modify the quality of the obtaining beer.

## 1. Introduction

In recent years, consumer interest in strong beers has been declining, while sales of low- and non-alcoholic beers have been increasing [1]. Additionally, customers are increasingly buying gluten-free beers or beers with reduced carbohydrate contents, which are characterized by the typical taste of traditional beers [2]. The main producers of low- and non-alcohol beers are large brewing industries. Therefore, small craft breweries, wanting to meet consumer expectations, create innovative non-alcoholic equivalents of their beers [3]. Nowadays, low- and non-alcohol beers are not just lagers with a weak taste and aroma, as a whole range of flavors and styles of NoLo (non-alcoholic and low-alcohol) beers are already available on the market. This improves the attractiveness of the available products for consumers with different preferences [4].

Among the many beer styles produced by craft breweries, wheat beers are very popular; however, mainly alcoholic ones are produced. For their production, wheat malt or unmalted wheat grain is used, replacing part of the barley malt [5]. Wheat beers are top-fermented beers characterized by a delicate taste, intense turbidity, and stable foam [6]. Due to its raw material composition, wheat beers contain a greater number of health-promoting substances, such as polyphenols, vitamins, trace elements, fiber, and antioxidants, with a relatively low ethanol content [7]. Antioxidants protect the body against oxidative stress, but they are sensitive to pH, temperature, oxygen levels, and light [8]. Craft breweries often produce drinks from unusual combinations of plant raw materials and beer, which affects their taste and aroma [9]. As in the case of wheat beers, fruit and spices are often used in their production [10]. For example, in the production of Belgian wheat beers, coriander seeds and orange peel are added [11]. Coriander seeds are used in beer production as a flavoring agent, which contributes to the formation of fruity and spicy notes [12]. Coriander (*Coriandrum sativum* L.) is a culinary and medicinal plant mainly cultivated in India [13]. Coriander seeds have a mild, sweet, slightly spicy, and citrus flavor with a hint of sage. The most important components of coriander seeds are essential oils and fatty oils [14]. Due to its properties, it is not only used in brewing but also as an additive in the production of other food products [15]. So far, very little research has been conducted on the impact of the addition of coriander seeds on the quality of the finished beer. An available literature report has focused on the addition of coriander only at the fermentation stage and its influence on the number of terpenes in the final product [16].

Therefore, the main aim of this research was to obtain low-alcohol wheat beers with the addition of coriander seeds at various stages (mash, boiling, and fermentation) of production. Before starting this research, the following research theses were formulated: the addition of coriander seeds at the mashing stage contributes to obtaining a final product characterized by a rich aromatic profile; and the addition of coriander seeds at the fermentation stage contributes to obtaining beers with greater antioxidant properties and an aromatic profile. The physicochemical properties and antioxidant activity of the finished beers were tested. Additionally, odor-active compounds were analyzed using a GC-MS instrument and GC-O gas chromatograph.

## 2. Results

### 2.1. Characteristics of Raw Materials—The Key Aroma Compounds of Coriander Seeds and Hops

**Figure 1:**
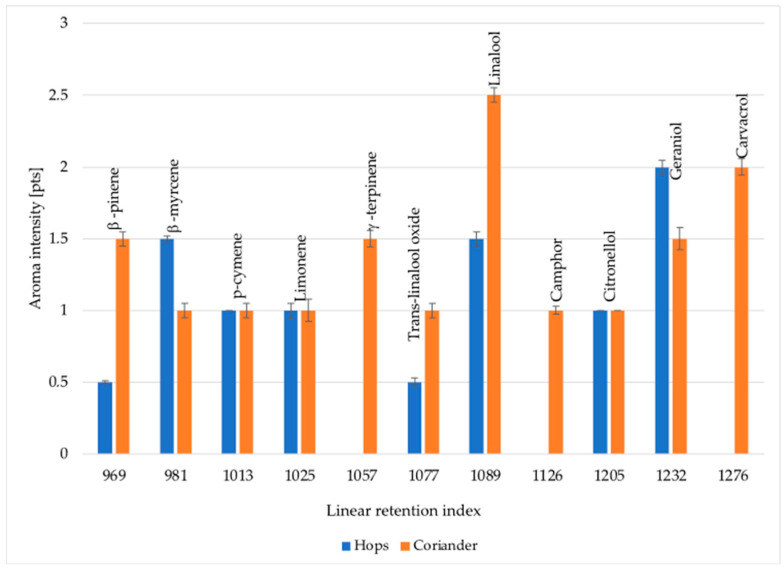
Odor-active compounds and their intensities in coriander and hops. n = 3; STD < 5%.

Odor-active components were determined in the raw materials used to produce wheat beer-rushed coriander seeds and hop. The Tettnanger variety of hops with an α-acid content of 5.5%, an aromatic variety typical for wheat beers, was used. The olfactometric analysis showed the presence of 11 odor-active aroma components in coriander seeds and 8 in the hops used (Table 1 and Figure 1). All the detected components were terpenes. γ-Terpinene, camphor, and carvacrol were the key aromas of coriander seeds that were not present in the hops used. Their content was 1232, 5646, and 65 μg/g, respectively. The olfactometric analysis (GC-O) also showed that the highest aroma intensity in the analyzed coriander seeds was found in linalool, carvacrol, and camphor, and their concentrations were above the detection thresholds. Particularly noteworthy was the 16 times higher concentration of linalool in the coriander seeds than in the hops. This accounted for over 70% of the content of all aroma components in the analyzed coriander seeds. A significantly higher geraniol content was also observed in the analyzed coriander seeds (2713 μg/g) compared to the analyzed hops (163 μg/g). In the case of hops, β-pinene and β-myrcene were characterized by the highest aroma intensity. These compounds are characterized by floral aromas, mainly rose. In the case of β-myrcene, a significantly higher amount was found in the analyzed hops (2394 μg/g) compared to coriander seeds (206 μg/g). This is also confirmed by the data presented by Takoi et al. [16], which describe that hops, regardless of their variety, are characterized by a higher content of this compound compared to coriander seeds.

### 2.2. The Fermentation Kinetics and Physicochemical Parameters of the Obtained Beers

**Figure 2:**
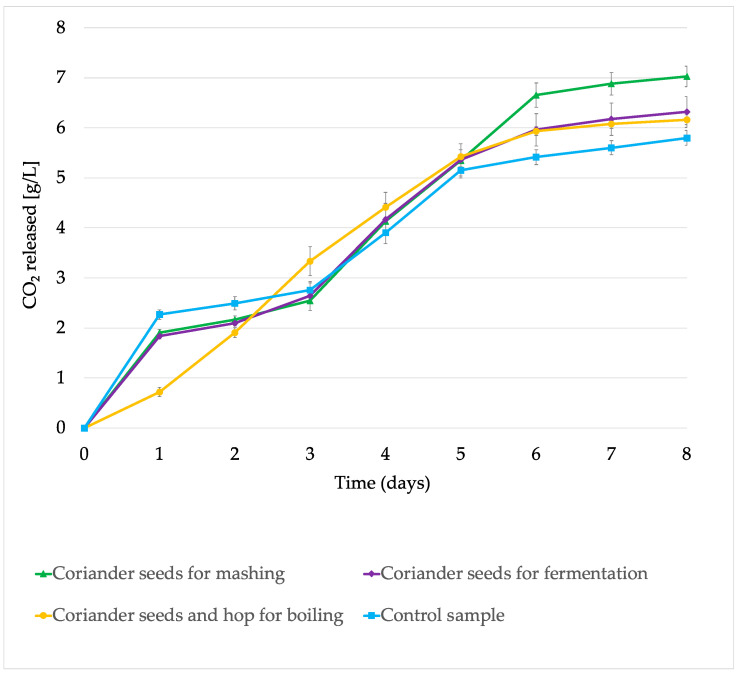
Fermentation kinetics of obtained beers n = 3; STD < 5%.

The kinetics of the fermentation process were measured from the day of inoculating the wort with a specific amount of yeast intended for the production of low- and non-alcohol beers (*Saccharomyces cerevisiae* var. *chevalieri*). This analysis was carried out until no changes were observed in the amount of CO_2_ (g/L) released on subsequent days of the process. As can be seen in Figure 2, on the first day of fermentation, the greatest amount of carbon dioxide was released from the control samples (without coriander). The lowest amount of CO_2_ released was observed in the case of samples with the addition of hops and coriander seeds during boiling. From the third to the fifth day of the process, all the samples fermented at a similar rate. From the sixth day of fermentation, the highest weight losses related to CO_2_ release were observed in samples to which coriander seeds were added during mashing. This was also confirmed by the alcohol content results (Table 2). The lowest final amount of carbon dioxide released was observed in the control samples without the addition of coriander seeds (Figure 2).

Wort obtained from barley and wheat malt was used for fermentation, which was characterized by a higher glucose and fructose content than maltose. This was achieved by modifying the mashing profile, i.e., omitting the saccharification pause at 62 °C. This composition of sugars made it possible to obtain low-alcohol beers (Table 2). The pH of the wort used for beer production was 5.9, and an antioxidant activity level of 125 mg/L was uncovered (Table 2). Table 2 also shows the basic physicochemical parameters of the finished beers. It was observed that the yeast used almost all the available glucose and fructose during fermentation. Beers produced with the addition of coriander seeds at the mashing stage were characterized by the highest amount of unattenuated maltose (6.61 g/L) compared to other samples. In turn, the addition of coriander seeds during fermentation contributed to obtaining beers with the highest antioxidant properties (142 mg/100 g of dry matter). The lowest antioxidant activity was observed in the case of control samples (without the addition of coriander seeds), but it was still higher than the antioxidant activity of unfermented wort (Table 2). The bitterness value of the beers was mainly influenced by the addition of hops; hence, the highest value of this parameter was obtained in the control sample. The addition of coriander seeds significantly reduced the bitterness of the beers; for example, if coriander seeds were introduced during the mashing or fermentation stages, the finished beers had an IBU value of 2.6 and 3.0, respectively. The resulting beer had an alcohol content of 1.3–1.7% *v*/*v*. The lowest alcohol content was found in the beers to which hops or coriander seeds were added during the boiling stage. In the case of these beers, a higher pH was also observed (4.7 and 4.6); however, these values are within the range for wheat beers, which was confirmed by the research conducted by Gugino et al. [18].

The quality of beer primarily depends on the raw materials, yeast strain, and technological process parameters. During fermentation, alcohol and carbon dioxide are mainly produced, along with numerous aroma compounds that make the composition of beers more complex than wort [19]. The presented article revealed the presence of 20 odor-active compounds, including 5 terpenes, 12 esters, and 3 other compounds. The most important group were the terpenes, which came from the raw materials used (coriander seeds, and hops). The highest concentrations were of linalool, camphor, and geraniol (Table 3), which were also present in the highest concentration in coriander seeds (Table 1). Linalool also showed high aroma activity, especially in beers to which coriander seeds were added during fermentation and boiling. Generally, all terpenes present in coriander seeds were also present in beers; however, not all were detected by the GC-O analysis, which was most likely due to their amounts being reduced below the detection limit. An example of such compounds was camphor, one of the key terpenes in coriander seeds (Table 4). The highest concentrations of terpenes occurred in beers to which coriander seeds were added during the fermentation or boiling stage. Of the eleven terpenes detected, seven had the highest concentrations in beers to which coriander seeds were added during the fermentation stage. At the same time, nine of them occurred at concentrations above the odor threshold, and three of them (linalool, β-pinene, and γ-terpinene) were detected during the GC-O analysis.

The second largest group of components in the beers were esters. All key aroma esters (Table 4) were detected in amounts above the aroma threshold. Of these, the largest amounts were ethyl acetate, isobutyl acetate, 1-butanol 3-methyl acetate, and ethyl hexanoate. The addition of coriander seeds had a statistically significant effect on the content of most esters. It turns out that they were present in the highest concentrations in the beers to which coriander seeds were added at the mashing or fermentation stage. The addition of coriander seeds at the fermentation stage again significantly influenced the aroma intensity of the esters because it was in these samples that most of the analyzed esters (ethyl acetate, isobutyl acetate, ethyl acetate, and ethyl decanoate), but also 2-phenylethanol, had the highest value of this parameter in the GC-O analysis. Among all esters, ethyl butyrate and ethyl valerate had the highest aromatic activity, regardless of the stage of adding coriander seeds during beer production.

In addition to terpenes and esters, there were also other key aroma compounds in the analyzed beers, such as acetophenone, decanal, and benzothiazoles. The concentration of these compounds was usually higher in the control beer without the addition of coriander seeds. However, their presence influenced the aroma because they were detected during the olfactometric analysis. All these components had the highest aroma intensity, mainly in the samples to which coriander seeds were added at the stages of fermentation and boiling.

**Figure 3:**
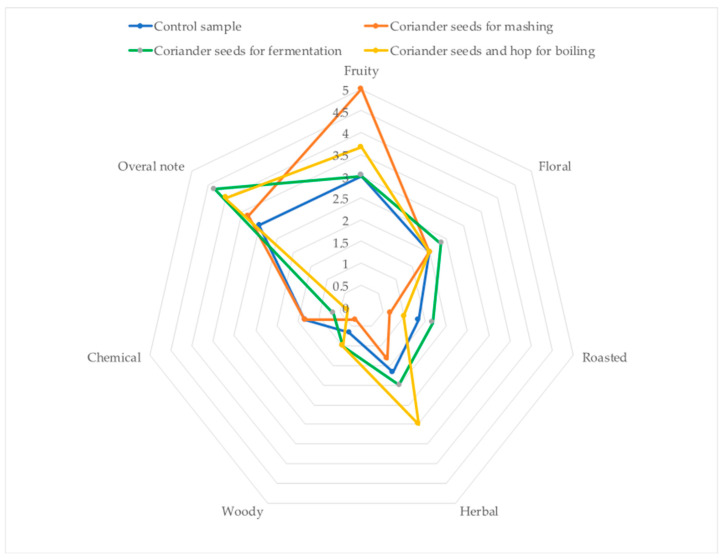
Sensory analysis (QDA) of produced beers with or without the addition of coriander seeds. n = 5; STD < 5%.

All four types of beers obtained were strongly differentiated through sensory analysis (QDA). The highest score (overall note) was given to the beers to which coriander seeds were added at the fermentation stage (almost 4.5/5 points); they also had the highest scores for floral, roasted, and woody aromas. The beers to which coriander seeds were added at the mashing stage received the highest notes for fruity, but also for chemical aroma, which resulted in a lower overall note (Figure 3). The most intense herbal and woody notes were detected in the beers with the addition of coriander seeds at the boiling stage. Samples without the added coriander seeds were rated the lowest in terms of all aroma characteristics (2.5/5 points), except for the chemical aroma.

## 3. Discussion

The taste and aroma of beer are the most important factors determining its sensory quality. Brewing raw materials, yeast species, and the parameters of the technological process significantly affect the aroma profile of beer. Malt introduces some flavor compounds into the wort, primally cereal, bread, caramel, or roasted. In turn, hops add fresh aromas to beer described as citrus, herbal, and fruity. Additionally, yeast produces a number of volatile compounds during fermentation, which also introduce fruity and floral notes [19]. For a very long time, brewing has used various herbs and additives in the production of beer to enhance the taste and aroma of the final product [20]. Coriander seeds are most often added to wheat beers due to their high content of essential oils, which give the beer a citrus aroma [11,12].

The presented article uses biological methods to produce low-alcohol (LAB) wheat beers with the addition of coriander seeds. For this purpose, first, the mashing process was modified (breaking at 44 °C for 20 min, then raising to 75 °C and maintaining it for 60 min). The main goal of modifying the mashing process is to inactivate β-amylase by mashing at high temperatures (72–80 °C) [21]. This contributes to obtaining a wort with a relatively high extract content, in which only about 25% are fermentable sugars [22]. Analyzing the sugar profile of the produced wort, it can be concluded that the modification of the mashing profile contributed to obtaining a significantly lower maltose content (the main sugar used by yeast in brewing) compared to other sugars such as glucose and fructose (Table 2). This value is approximately 90% lower than the results obtained by Zdaniewicz et al. [23] during standard mashing (the longest break at a temperature of 70 °C). The second method used to obtain LAB is the selection of an appropriate yeast culture with limited ability to ferment sugars from the wort (e.g., maltose and maltotriose) [4]. In the presented studies, wort with a low maltose content was inoculated with the yeast *Saccharomyces cerevisiae* var. *chevalieri*, which was intended for the production of LAB. This yeast does not assimilate maltose and maltortriose and is characterized by a subtle aroma profile. Therefore, the composition of the wort has a great influence on the metabolism of yeast during the production of low-alcohol beers [24]. The yeast used in the presented article used almost all the available glucose and fructose. This contributed to obtaining beers with alcohol contents in the range of 1.3–1.7% *v*/*v* (Table 2). These results are similar to the research conducted by Dziedziński et al. [25], where the same yeast strain was used to produce low-alcoholic IPA beer. In the presented article, the control sample (beers hopped while boiling) had the lowest alcohol content, which is also confirmed by its fermentation kinetics (Figure 1). The lower alcohol content in this case could be caused by the higher content of iso-α-acids in hopped beers (bitterness at the level of 25–30 IBU units), which influenced the metabolism of the yeast. This is also confirmed by the research conducted by Yang et al. [26], where a higher level of iso-α-acids reduced the formation of ethanol during fermentation by some yeast strains. Additionally, no negative impact of the addition of coriander seeds on the beer fermentation process was observed. The use of specific yeast strains for the production of low- and non-alcohol beers provides an opportunity to keep the initial flavor profile of the beer intact. This method has an advantage over physical methods, where after the dealcoholization process, alcohol-free beer is characterized by a weakened aromatic profile [4].

Despite many studies on the raw materials used to produce beer and the technological process itself, there are few reports on the impact of individual additives on its antioxidant activity. The antioxidant activity and profile of polyphenols and phenolic acids vary depending on the type of beer. Typically, the highest total phenol content values are found in fruit and dark beers, and the lowest is found in non-alcoholic beers [27]. Adding coriander seeds to beer can increase its antioxidant effects. Coriander seeds contain significant amounts of antioxidants and essential oils, which may provide antibacterial, antifungal, and antioxidant effects [28]. Beers with the addition of coriander seeds during fermentation had the highest antioxidant properties (142 mg/10 g dry mass), and this parameter increased by approximately 14% compared to the wort. This may indicate greater thermal stability of these compounds derived from coriander seeds. Previous research has confirmed that the addition of coriander seeds during fermentation has a positive effect on the antioxidant activity of the finished kombucha product [29]. The addition of coriander seeds to non-alcoholic beer introduces some terpenes. Terpenes, in addition to the aroma, could have anti-inflammatory properties (e.g., limonene, linalool, and α-pinene) and cytotoxic activity (e.g., limonene, linalool, and geraniol), and there have been various studies investigating such an activity against cancer cells [30].

As already mentioned, most low- and non-alcohol beers available on the market have a weak aroma profile due to their production method. Breweries using physical methods to remove alcohol content in the production of such beers face the greatest challenge of retaining all volatile compounds, affecting the quality of the finished product [31]. The taste and aroma of beer is the result of the complex interaction of hundreds of chemical compounds and their perception by olfactory and taste receptors [32]. The quality of the finished product depends not only on the type of yeast used for its production but also on the share of individual raw materials and the technological process underlying their production [33,34]. So far, no research has been carried out on the analysis of volatile compounds during the addition of coriander seeds at various stages of low-alcohol beer production. The available research focuses on the changes that occur during the addition of coriander seeds during fermentation [16]. This current article, however, undertakes a comparative analysis of volatile compounds in beers with the addition of coriander seeds at various stages of their production (mashing, boiling, and fermentation). Crushed coriander seeds were added in the amount of 150 g/1 hL of beer, in accordance with the recommendations of technologists/brewers. The control sample was beer without the addition of coriander seeds (beer with the addition of hops during boiling). The results presented by Jeleń et al. [35] confirm the occurrence of linalool, geraniol, and camphor in coriander oil. In our research, typical aromatic hops for the production of wheat beers (Tettnanger 5.5% alpha acids) were also used as an addition. These hops, in turn, was characterized by a high content of β-myrcene and geraniol. In the specification, the manufacturer confirms the presence of myrcene, which constitutes an average of 20–35% of total oil [36].

The most important group of compounds originating from the raw materials used (coriander seeds and hops) found in the obtained beers were terpenes (11 different compounds were detected). According to the literature, terpenes that give beers their important organoleptic properties come mainly from hops. Some of them are metabolized by yeast during the fermentation process into other terpenoid compounds [37]. Monoterpene alcohols, including linalool and geraniol, have a very floral aroma and significantly shape the bouquet of alcoholic beverages [38]. Analyzing individual variants of the obtained beers, it was observed that the addition of coriander seeds during fermentation contributed to obtaining beers with the highest terpene content compared to the other samples (Table 3). A significant content of linalool, β-pinene, γ-terpinene, camphor, geraniol, and carvacrol was observed in these beers compared to the control sample and other variants. All of the above-mentioned compounds were present in significant amounts in the coriander seeds used in this research. These compounds entered the beer as a result of the contact between the crushed coriander seeds and beer during fermentation. The process of adding coriander seeds during the fermentation stage is similar to dry hopping (addition of hops at the fermentation stage). Dry hopping involves the extraction of volatile/non-volatile hop compounds in an alcohol solution [39]. The coriander seeds used were characterized by a high content of individual oils, which is confirmed by the results obtained (Table 1). The extraction and solubility of compounds derived from seeds increased with increasing alcohol content [40]. In the studies conducted by other scientists, it was also observed that during fermentation, compounds such as linalool and geraniol are transferred from coriander seeds into the finished beer [41,42], which is also confirmed by our research. A large amount of linalool has a significant impact on the aroma of the obtained beers, which is confirmed by the results of our olfactometric analyzes (Table 4). As a result, it can be concluded that the addition of coriander seeds during the fermentation stage can enhance the citrus/floral/fruity character of the resulting products. Additionally, according to the literature data, the addition of hops during the fermentation stage is not very profitable because about two-thirds of the dry matter of hops are wasted, regardless of the variety used [39]. Therefore, the addition of coriander seeds during this stage is a good solution because it enriches the beer with the appropriate terpenes without causing too much of a loss of raw materials. Interesting results were also obtained in the case of beers with the addition of coriander seeds during boiling together with hops. Significantly higher contents of linalool, β-myrcene, and citronellol were observed compared to the control sample. These compounds are characterized by citrus, floral, and rose aromas [43], which are confirmed by the results obtained after our olfactometric analysis. In the research conducted by Takoi [16], the content of individual terpenes was analyzed after boiling the wort with various hops together with coriander during their production of Belgian-style white beers. Among the detected terpenes, linalool, β-myrcene, and citronellol predominated. In turn, coriander seeds during the mashing stage contributed to obtaining beers with the weakest aromatic profile. It was observed that this beer was characterized by a significantly higher content of trans-linalool oxide compared to the control sample (Table 3). This compound entered the beer during the stage of mashing from the coriander seeds, which contained it in significant amounts (Table 1). Further compounds that entered the beer during the mashing stage and came from coriander seeds were camphor and carvacrol. It is worth noting that these compounds were not detected at all in the control sample. During mashing, aroma compounds from the raw materials used (mainly malt) are extracted from the malt grits and transferred into the wort [44]. Additionally, a modified mashing profile (mash at a higher temperature) could have contributed to the transfer of compounds found in coriander seeds into the finished beer. Research related to the addition of coriander seeds during the mashing stage can be extended to include various doses of the raw material. In the present study, only one concentration of coriander seeds was used to check whether changes would be observed. This is even more interesting because research has already been carried out on the so-called hopping the mash (adding hops to the mash), where satisfactory results were not obtained. Kolbach and Wilhram [45] found large losses of the aroma components. A similar phenomenon was observed by Schur and Pfenninger [46]; however, at the same time, they found a more delicate and distinct hop aroma in the finished beers. Additionally, after the olfactometric analysis, it was found that these beers did not have the key coriander seed aromas. Compared to other variants, they are characterized by the weakest aromatic profile. Therefore, further research is needed to ensure that the addition of coriander seeds during the mashing stage contributes to increasing the level of aroma in the finished product. The second largest group of volatile compounds in the analyzed beers were esters (especially ethyl acetate, ethyl hexanoate, ethyl octanoate, and ethyl decanoate). These compounds were also found in non-alcoholic wheat beers produced by biological methods that have been presented in various scientific studies [47]. Esters are formed in beer during fermentation as a result of the enzymatic condensation of organic acids and alcohols. Esters play a key role in the taste of non-alcoholic beer, introducing fruity and floral aromas [27]. The aroma of ethyl acetate is described as floral; ethyl hexanoate has a fruity-like red apple aroma; ethyl octanoate gives sweet, fruity, and winey aromas; and ethyl decanoate provides sweet, fruity, and brandy aromas [17]. Beer production based on biological methods allows us to obtain high aromatic beers, which was confirmed by our olfactometric analysis. In the analyzed beers, the highest amount of esters was observed in the beers to which coriander seeds were added during the mashing stage. This is also confirmed by the results of the sensory analysis that we carried out, during which the samples with the addition of coriander (regardless of the stage of their addition) received a higher score compare to the control. Lutosławski et al. [48] also confirmed that the addition of coriander significantly positively intensified the aroma in Witbier-style beer.

## 4. Materials and Methods

### 4.1. Materials

For malt production, commercially available pilsner malt and wheat malt were used (Viking Malt, Strzegom, Poland), along with the hop variety Tettnanger, 5.5% alpha acids (Yakima Valley Hops, USA). During beer production, coriander seeds (Prowana Sp. z o. o. Radzymin, Poland) were added (150 g/hL) during various technological stages (mashing, boiling, and fermentation). Before adding the coriander seeds, they were sterilized using UV radiation. For low-alcoholic beer production, the *Saccharomyces cerevisiae* var. *chevalieri* (Fermentis SafBrew LA-01, Poland) was used.

### 4.2. Beer Production

#### 4.2.1. Milling, Mashing, and Lautering

Malt mashes were prepared in Mash Bath R12 (1-CUBE, Czech Republic). The malt was milled in a disc mill, weighed into tarred mash containers, and placed in a water-heated apparatus at 44 °C, where it was held for 20 min. During this stage, an appropriate dose of coriander was added to some of the variants. The mash was then heated to 75 °C and held for 60 min. The chosen mashing profile was previously conceived and checked by the authors of this manuscript. The resulting beers were characterized by appropriate quality parameters, so it was decided to use them for the production of low-alcohol wheat beers. Next, the containers were cooled to 20 °C, filled with distilled water up to a mass of 450.0 g, and filtered through a paper filter (MN614). To ensure high clarity, the first portions of the filtrate were recirculated.

#### 4.2.2. Boiling

The obtained wort was divided into individual variants and subjected to the boiling process. Variants I and II involved boiling the wort without any additives. Variant III involved boiling the wort with the addition of Tettnanger hops (5.5% alpha acids) and crushed coriander seeds (150 g/hL). Variant IV was boiled with the addition of Tettnanger hops (5.5% alpha acids). All variants were boiled for 60 min, after which the hot tub was removed from the wort using Whatman class 802 filter paper. Then, all the variants were cooled to 20 °C and brought to a common extract (9 °P). Before inoculation, all the most important quality parameters of the obtained wort were analyzed.

#### 4.2.3. Fermentation Trials

After boiling, all the prepared samples were inoculated with yeast cells (*Saccharomyces cerevisiae* var. *chevalieri*) to obtain live yeast cell suspensions of 5 × 10^6^ cfu/mL based on the wort extract. The number of yeast cells in 1 mL of suspension was calculated using a Thoma chamber. Sterilized coriander seeds were added to 3 repetitions in the same amount as in the previous stages (150 g/hL). Then, all of the samples (250 mL) were fermented in Erlenmeyer flasks (500 mL) under anaerobic conditions (plugs with fermentation tubes filled with glycerine) at 20 °C for 8 days (Q-CELL 240 thermostatic chamber, Wilkowice, Poland). The kinetics of fermentation were monitored by measuring the mass losses related to the release of carbon dioxide (g/L) over the consecutive days of the process.

### 4.3. Analytical Determinations

#### 4.3.1. The International Bitterness Unit (IBU)

The analysis of the content of iso-α-acids in the obtained beers was performed using the isooctane extraction method from the acidified samples. After extraction, absorbance analysis was performed (Beckman DU-650 UV-Vis, East Lyme, CT, USA) at a wavelength of 275 nm according to the Analytica EBC method 8.8 [49].

#### 4.3.2. Ethanol and Real Extract

These analyzes were measured with an automatic beer analyzer (Alcolyzer, Anton Paar DMA 4500+, Warsaw, Poland). The samples were degassed by mixing beer with diatomaceous earth and filters through filter paper to obtain 50 mL of filtrate. The filtrate was degassed for 20 min (universal shaker, 150 rpm), brought to 20 °C, and filtered again.

#### 4.3.3. The pH of the Obtained Wort and Beer

The pH of the wort and beers was measured using the Mettler Toledo FiveGo pH meter (Warsaw, Poland).

#### 4.3.4. The Color of the Obtained Wort and Beer

The filtered wort and beers were measured spectrophotometrically (Beckman DU-650 UV–Vis, East Lyme, CT, USA) at a wavelength of 430 nm according to the Analytica EBC method [50].

#### 4.3.5. The Turbidity of the Obtained Wort and Beer

Wort and beer turbidity were measured with a nephelometer Cyberscan TN 100 (Merazet, Poznań, Poland), which applied the nephelometric method in the range of 0–1000 NTU, according to EN/ISO 7027, in order to measure the level of turbidity.

#### 4.3.6. Antioxidant Activity (AOX)

Antioxidant activity (AOX) was determined according to the method described by Tarko et al. [51]. For this purpose, the active cationic radical 2,2′-azino-bis(3-ethylbenzothiazole-6-sulfonic) acid (ABTS, Sigma-Aldrich) was used. Antioxidant activity was calculated based on the calibration curve and expressed in mg Trolox/100 mL.

#### 4.3.7. Sugar Analysis Using a High-Performance Liquid Chromatograph (HPLC)

Our sugar analysis was conducted in accordance with Satora and Pater [52] using the Shimadzu (Kyoto, Japan) NEXERA XR apparatus with an RF-20A refractometric detector. The separation was conducted with an Asahipak NH2P-50 4.6 × 250 mm Shodex column (Showa Denko Europe, Munich, Germany) thermostated at 30 °C. The mobile phase consisted of an acetonitrile aqueous solution (70%), and the isocratic elution program (0.8 mL/min) lasted for 16 min. Quantitative determinations were made with the use of standard curves prepared with the appropriate standards: glucose, fructose, and maltose (Sigma-Aldrich).

#### 4.3.8. Odor-Active Volatile Components (HS-SPME-GC-O)

Odor-active volatile compounds of beers were identified by olfactometry. A two mL sample of beer with 1 g of NaCl placed in a 10 mL vial was exposed to a 50/30 µm divinylbenzene/carboxen/polydimethylsiloxane (DVB/CAR/PDMS) SPME fiber (Supelco/Sigma-Aldrich, Bellafonte, PA, USA) for 40 min at 40 °C. Subsequently, the SPME device was introduced into the injector port of the Hewlett Packard 5890 Series II chromatograph system and kept in the inlet for 3 min. The tested components were separated on a Rxi^®^-1ms capillary column (Crossbond 100% dimethyl polysiloxane; 30 m × 0.53 mm × 0.5 m). The detector temperature was 250 °C, and the column was heated using the following program: 35 °C for four minutes at an increment of 5 °C/min to 110 °C, then an increment of 20 °C/min to 230 °C, and then maintaining a constant temperature for 4 min. The carrier gas was helium at a 1.0 mL/min constant flow. For this analysis, an olfactory detection port (ODP-3, Gerstel, USA) was used. Three trained GC-O analysts were asked to describe the odors they perceived and their intensity. The method of odor intensity was used with a 4-point scale (not detected, weak, moderate, and strong). For each beer sample, the odor-active compounds were identified and grouped into classes according to their chemical functional group. The sums of odor intensities for each class of compounds were calculated. 

#### 4.3.9. Analysis of Volatile Compounds Using HS-SPME-GC-MS

In order to analyze the volatile compounds, 2 mL of beer and 0.1 mL of an internal standard solution (0.57 mg/L 4-methyl-2-pentanol, 0.2 mg/L anethol, and 1.48 mg/L of ethyl nonanoate (Sigma-Aldrich, Saint Louis, MO, USA)) were placed into a 10 mL vial with 1 g of NaCl. The conditioned (250 °C for 1 h) SPME device (Supelco Inc., Bellefonte, PA, USA) coated with PDMS (100 µm) fibers was used for sampling. It was placed into the headspace under stirring (300 rpm) for 40 min at 40 °C. Next, the SPME device was desorbed in the injector port of the chromatograph system for 3 min. The Shimadzu GC-2010 Plus gas chromatograph coupled with the GCMS-QP2020 system was used for these analyzes. A Rxi^®^-1 ms capillary column (Crossbond 100% dimethyl polysiloxane; 30 m × 0.53 mm × 0.5 µm) was used for the separation of the analyzed volatiles. The column was heated using the following program: 35 °C for 4 min at an increment of 5 °C/min to 110 °C, then an increment of 20 °C/min to 230 °C, and then maintaining a constant temperature for 4 min. Helium was the carrier gas at a constant flow of 1.0 mL/min. The mass spectra were recorded in the SIM mode at an ionization voltage of 70 eV and a transfer line and ion source temperature of 250 °C. Analytes were transferred in the splitless mode. The mass spectrometer detector (MSD) was set to the scan mode from *m*/*z* = 40 to *m*/*z* = 400.

Volatiles were identified using the National Institute of Standards and Technology (NIST) database and LRIs (linear retention indices), calculated based on a series of n-alkanes from C6 to C30. The quantitative identification of volatile compounds (Sigma-Aldrich) was based on the comparison between the peak surface area of the sample and the standard chromatograms.

#### 4.3.10. Sensory Assessments

The sensory analysis of the obtained beers was based on their aromas and included six sensory descriptors (fruity, floral, roasted, herbal, woody, and chemical) rated on a 5-point hedonistic scale in quantitative descriptive analysis (QDA). The panelists were scientific staff working in the Faculty of Food Technology and Human Nutrition (University of Agriculture in Krakow) who previously graduated from that faculty and obtained an extensive course on sensory analysis as a part of their curriculum. First, the panelists received standards of different aromas, checking whether they were able to recognize each of them. Then, they received the same standards but at various concentrations. Only those who passed those two stages were selected as panelists. Beers were subjected to sensory assessments by the panel comprising 10 panelists. The samples were coded and provided to the panelists in a randomized order. The results were subjected to the one-way analysis of variance (ANOVA).

## 5. Conclusions

In conclusion, this study indicates that the addition of coriander seeds during beer production positively influences its sensory quality and exhibits an antioxidant activity. The most significant improvements were observed when the coriander seeds were added during the fermentation stage, resulting in the production of beers with the most favorable aromatic profile. This is particularly noteworthy, as it suggests a positive outcome for reducing hop losses during dry hopping. The extended contact time between the coriander seeds and beer during the lengthy fermentation stage likely contributes to the enhanced aromatic characteristics. On the other hand, beers with coriander seeds added during the mashing stage displayed a less robust aromatic profile. Despite this, this study observed some transfer of compounds from the coriander seeds into the beers during this stage. As a result, future research has been planned to explore the potential impact of increasing the coriander seed dosage to assess whether it will have any notable effects on the aromatic profile of the beers. This indicates a promising avenue for further investigation and potential optimization of the beer production process. Taking into account all the results obtained, the use of coriander seeds in the production of low- and non-alcohol beers seems to be the right way for increasing the level of full flavor and aromaticity.

## Figures and Tables

**Table 1:** Odor-active compounds of coriander seeds and hops.

(μg/g)	*m*/*z*	LRI ^2^	Threshold ^3^	Coriander Seeds	Hops (Tettnanger 5.5 % Alpha Acids)	Sig ^1^	GC-O Descriptors ^4^
Terpenes							
β-pinene	41, 69, and 93	969	4	85 ^a^	203 ^b^	**	Pine and coriander [H]
β-myrcene	41, 69, and 93	981	350	206 ^a^	2394 ^b^	***	Floral [FL]
p-cymene	91, 119, and 134	1013	55.7	2	4	ns	Citrus [FR]
Limonene	68, 79, and 93	1025	65	343 ^a^	74 ^b^	**	Lemon and citrus [FR]
γ-terpinene	91, 93, and 136	1057	25	1232 ^a^	0 ^b^	**	Floral and coriander [FL]
Trans-linalool oxide	43, 59, and 94	1077	5	295	229	ns	Flower [FL]
Linalool	55, 71, and 93	1089	6	27,934 ^a^	1706 ^b^	***	Floral, lavender, and lille [FL]
Camphor	41, 81, and 95	1126	80	5646 ^a^	0 ^b^	***	Woody [W]
Citronellol	41, 67, and 69	1205	3.55	97 ^a^	16 ^b^	**	Rose [FL]
Geraniol	41, 69, and 93	1232	4	2713 ^a^	163 ^b^	**	Rose and geranium [FL]
Carvacrol	90, 135, and 150	1276	2900	65 ^a^	0 ^b^	***	Grassy and herbaceous [H]

^1^ Significance; ** and *** indicate significance at a level of 0.01–0.005 and <0.005, respectively, by the least significant difference. Values with different superscript roman letters (a,b) in the same row indicate statistical differences according to the Duncan test (*p* < 0.05). ^2^ LRI—linear retention index; the amount of components was determined. ^3^ Threshold in beer [17]. OAV > 1. ^4^ Aroma descriptor perceived at the sniffing port of the GC-O. The aroma group of the detected aroma descriptors was signed by letters in brackets—fruity (FR), floral (FL), herbaceous (H), and woody (W). SD < 5%.

**Table 2:** The main parameters of beer produced with the addition of coriander.

Parameter	Extract (°P)	Color (EBC Units)	Turbidity (EBC Units)	pH	Ethanol (% *v*/*v*)	Real Extract (% *w*/*w*)	Bitterness (IBU Units)	Antioxidant Activity (mg/100 mL)	Glucose (g/L)	Fructose (g/L)	Maltose (g/L)
Wort	9.0 (±0.0)	10.3 ^a^ (±1.28)	17.6 ^a^ (±1.53)	5.9 ^a^ (±0.0)	-	-	-	125 ^a^ (±0.02)	51.8 ^a^ (±7.93)	10.8 (±1.18)	7.62 (±1.81)
Control sample	-	11.3 ^a^ (±1.99)	115 ^b^ (±177.8)	4.6 ^c^ (±0.21)	1.3 ^a^ (±0.05)	5.0 ^b^ (±0.28)	30 ^c^ (±0.52)	130 ^b^ (±0.52)	0.14 ^b^ (±0.02)	0.22 ^b^ (±0.02)	2.41 ^b^ (±1.4)
Coriander seeds for mashing	-	20.8 ^b^ (±5.35)	109 ^b^ (±102.1)	4.2 ^bc^ (±0.19)	1.7 ^b^ (±0.13)	4.3 ^a^ (±0.18)	2.6 ^a^ (±0.02)	135 ^b^ (±0.08)	0.08 ^b^ (±0.03)	0.26 ^b^ (±0.04)	6.61 ^a^ (±1.04)
Coriander seeds for fermentation	-	25.7 ^b^ (±4.52)	120 ^b^ (±194.2)	3.8 ^b^ (±0.14)	1.5 ^ab^ (±0.07)	4.6 ^ab^ (±0.11)	3.0 ^a^ (±0.25)	142 ^c^ (±0.25)	0.16 ^b^ (±0.02)	0.15 ^b^ (±0.05)	4.87 ^b^ (±1.83)
Coriander seeds and hop for boiling	-	20.4 ^b^ (±4.7)	154 ^b^ (±23.7)	4.7 ^c^ (±0.38)	1.4 ^a^ (±0.12)	4.7 ^ab^ (±0.10)	25 ^b^ (±0.97)	138 ^bc^ (±0.01)	0.14 ^b^ (±0.05)	0.21 ^b^ (±0.05)	2.54 ^b^ (±1.97)
^1^ Sig	-	*	*	***	*	*	***	**	***	***	**

^1^ Significance; *, **, and *** indicate significance at a level of 0.01–0.005 and <0.005, respectively, by the least significant difference. Values with different superscript roman letters (a–c) in the same column indicate statistically differences according to the Duncan test (*p* < 0.05).

**Table 3:** Odor-active compounds of beers with the addition of coriander at individual stages of its production.

(μg/L)	*m*/*z*	LRI ^2^	Threshold ^3^	Control Sample	Coriander Seeds for Mashing	Coriander Seeds for Fermentation	Coriander Seeds and Hop for Boiling	Sig ^1^	GC-O Descriptors ^4^
**Alcohols**									
3-methyl-1-butanol	42, 55, and 70	716	1000	67,970 ^a^	82,495 ^ab^	88,412 ^b^	68,531 ^ab^	*	Bready, alcoholic, and fruity [R]
2-methyl-1-butanol	41, 57, and 70	724	15.9	21,189	21,001	20,966	27,719	ns	Malt and sweet [FR]
2-phenylethanol	91, 65, and 122	1091	1000	815	1325	1090	799	ns	Rose [FL]
**Esters**									
Ethyl acetate	43, 61, and 70	598	5000	27,277 ^a^	43,318 ^b^	46,379 ^b^	33,029 ^a^	**	Floral and solvent [FL]
Isobutyl acetate	43, 56, and 73	758	100	87.7 ^a^	2119 ^b^	177 ^a^	131 ^a^	**	Fruit, apple, and banana [FR]
Ethyl butyrate	43, 71, and 88	784	150	99 ^a^	207 ^b^	118 ^a^	118 ^a^	**	Pineapple, sweet, and fruity [FR]
Ethyl 2-methylobutyrate	41, 57, and 102	836	0.3	12	13	18	15	ns	Fruity, apple, and peach [FR]
1-butanol 3-methyl acetate	43, 55, and 70	860	220	1397	1644	1029	1656	ns	Fruity and apple [FR]
Ethyl valerate	57, 85, and 88	883	1	8 ^a^	9 ^a^	13 ^b^	6 ^a^	*	Yeast and fruit [FR]
Ethyl hexanoate	43, 88, and 99	980	5	546 ^a^	1417 ^b^	1428 ^b^	538 ^a^	**	Fruity and red apple [FR]
Ethyl octanoate	57, 88, and 101	1179	70	747	1005	1010	747	ns	Sweet, fruity, and winey [FR]
Ethyl decanoate	43, 88, and 10	1373	200	927	1266	1081	941	ns	Sweet, fruity, and brandy [FR]
**Terpenes**									
β-pinene	41, 69, and 93	969	4	1 ^a^	1 ^a^	14 ^b^	5 ^ab^	*	Pine and coriander [H]
β-myrcene	41, 69, and 93	981	13	223 ^a^	5 ^b^	15 ^b^	1492 ^c^	**	X
p-cymene	91, 119, and 134	1013	55.7	1 ^a^	1 ^a^	6 ^b^	13 ^c^	**	Citrus [FR]
Limonene	68, 79, and 93	1025	65	8 ^a^	2 ^a^	14 ^b^	63 ^c^	**	Lemon and citrus [FR]
γ-terpinene	91, 93, and 136	1057	25	2 ^a^	3 ^a^	46 ^b^	25 ^b^	*	Floral and coriander [FL]
Trans-linalool oxide	43, 59, and 94	1077	5	70 ^a^	75 ^a^	369 ^b^	274 ^c^	**	X
Linalool	55, 71, and 93	1089	6	1112 ^a^	484 ^b^	0 ^c^	7416 ^d^	***	Flower and lavender [FL]
Camphor	41, 81, and 95	1126	80	0 ^a^	49 ^a^	2721 ^b^	1239 ^c^	***	X
Citronellol	41, 67, and 69	1205	8	209 ^a^	11 ^b^	163 ^a^	272 ^a^	**	Rose [FL]
Geraniol	41, 69, and 93	1232	4	203 ^a^	19 ^b^	439 ^c^	365 ^d^	***	X
Carvacrol	90, 135, and 150	1276	2900	0.9 ^a^	7 ^b^	12 ^b^	7 ^b^	**	X
**Others**									
Acetophenone	51, 77, and 105	1036	65	12 ^a^	4 ^b^	4 ^b^	4 ^b^	**	Sweet, pungent, and chemical [C]
Decanal	41, 43, and 57	1182	0.1	26 ^a^	24 ^a^	0 ^b^	30 ^a^	**	Aldehydic, citrus, and floral [FL]
Benzothiazole	69, 108, and 135	1196	80	197	184	153	158	ns	Gasoline and rubber [C]

^1^ Significance; ns—not statistically; *, **, and *** indicate significance at a level of 0.05–0.01, 0.01–0.005, and <0.005, respectively, by the least significant difference. Values with different superscript roman letters (a–c) in the same raw indicate statistical differences according to the Duncan test (*p* < 0.05). ^2^ LRI—linear retention index; the amount of components was determined. ^3^ Threshold in beer [17]. OAV > 1. ^4^ Aroma descriptor perceived at the sniffing port of the GC-O. X—not detected in the GC-O analysis. Aroma group of detected aroma descriptors was signed by letters in brackets— roasted (R), fruity (FR), floral (FL), herbaceous (H), and chemical (C). SD < 5%.

**Table 4:** Heatmap of odor-active compounds intensities detected by GC-O in beers produced with the addition of coriander.

Compound	^1^ LRI	Control Sample	Coriander Seeds for Mashing	Coriander Seeds for Fermentation	Coriander Seeds and Hop for Boiling
3-methyl-1-butanol	716	0.5	0.8	1.0	1.0
2-methyl-1-butanol	724	1.3	0.8	1.0	1.2
2-phenylethanol	1091	0.0	0.5	1.2	0.6
Ethyl acetate	598	1.0	0.6	1.2	0.3
Isobutyl acetate	758	0.6	0.0	1.0	1.0
Ethyl butyrate	784	0.6	0.8	1.0	1.4
Ethyl 2-methylobutyrate	836	0.6	0.8	0.6	0.8
3-Methylbutyl acetate1-butanol 3-methyl acetate	860	0.6	1.0	0.6	0.6
Ethyl valerate	883	1.3	0.8	1.0	1.2
Ethyl hexanoate	980	1.3	1.3	1.0	0.3
Ethyl octanoate	1179	1.0	0.0	1.3	1.2
Ethyl decanoate	1373	1.0	0.2	1.2	0.3
β-pinene	969	0.0	0.6	1.2	0.6
Limonene	1025	0.6	0.0	0.3	0.0
γ-terpinene	1057	0.0	0.3	1.0	1.0
Linalool	1089	0.6	0.8	1.6	0.6
Citronellol	1205	0.6	0.0	0.3	0.0
Acetophenone	1036	0.8	0.3	1.2	1.0
Decanal	1182	0.0	0.8	0.3	0.0
Benzothiazole	1196	0.8	0.3	1.0	0.0

^1^ LRI—linear retention index. The lowest intensity of aromas in these columns is in the darkest red, and the highest intensity is in the darkest green. SD < 5%.

## Data Availability

Data are contained within the article.
